# De Novo Primary Squamous Cell Carcinoma of the Prostate: Substantial Tumor Regression After Definitive Radiotherapy in a Medically Inoperable Patient

**DOI:** 10.3390/life16050702

**Published:** 2026-04-22

**Authors:** Sang Jun Byun, Misun Choe, Jin Young Kim, Byung Hoon Kim, Hyun Chan Jang, Seung Gyu Park, Euncheol Choi, Sang Hee Youn, Myeongsoo Kim, Byungyong Kim, Byungwook Choi

**Affiliations:** 1 Department of Radiation Oncology, Keimyung University School of Medicine, Daegu 42601, Republic of Korea; psk818@dsmc.or.kr (S.G.P.); cec0510@dsmc.or.kr (E.C.); mskim@dsmc.or.kr (M.K.); 2 Department of Radiation Oncology, Keimyung University Dongsan Hospital, Daegu 42601, Republic of Koreafacorn@dsmc.or.kr (B.K.); 3 Department of Pathology, Keimyung University School of Medicine, Daegu 42601, Republic of Korea; msc@dsmc.or.kr; 4 Department of Internal Medicine, Keimyung University School of Medicine, Daegu 42601, Republic of Korea; takgu@dsmc.or.kr; 5 Department of Urology, Keimyung University School of Medicine, Daegu 42601, Republic of Korea; blackporori@dsmc.or.kr; 6 Department of Urology, Keimyung University Dongsan Hospital, Daegu 42601, Republic of Korea; hcjang@dsmc.or.kr; 7 Department of Nuclear Medicine, Daegu Catholic University School of Medicine, Daegu 42472, Republic of Korea; nmchoibw@gmail.com

**Keywords:** primary prostatic squamous cell carcinoma, de novo, definitive radiotherapy, medically inoperable, locally advanced disease

## Abstract

Primary squamous cell carcinoma (SCC) of the prostate is a rare and biologically aggressive malignancy lacking a standardized management strategy. De novo primary SCC arising without prior androgen deprivation therapy or radiotherapy is uncommon and presents significant diagnostic and therapeutic challenges. We present the clinical presentation, diagnostic evaluation, treatment strategy, and early therapeutic response of de novo primary SCC of the prostate in a 56-year-old male with end-stage renal disease on maintenance hemodialysis. The patient presented with gross hematuria and a bulky prostate mass invading the bladder with bilateral pelvic lymphadenopathy despite low prostate-specific antigen (PSA) levels. Histopathological and immunohistochemical analyses confirmed pure SCC, staged as cT4N1M0. Because systemic chemotherapy was contraindicated and surgery was not feasible, definitive whole-pelvis radiotherapy with a simultaneous integrated boost was administered. Marked tumor regression was observed one month after treatment. Subsequent imaging demonstrated extensive tumor necrosis with fistulous communication in the context of locally invasive disease. Because long-term oncologic durability could not be assessed owing to non-oncologic clinical deterioration, these findings suggest that definitive radiotherapy may provide meaningful locoregional tumor control in selected medically inoperable patients with de novo prostatic SCC.

## 1. Introduction

Prostate cancer is predominantly adenocarcinoma, whereas primary squamous cell carcinoma (SCC) of the prostate is an exceptionally rare histological subtype, representing <1% of all prostatic malignancies [1]. Due to its rarity, the biological behavior, optimal treatment strategy, and clinical outcomes of prostatic SCC remain poorly understood, and evidence-based treatment guidelines have not yet been established [2].

Compared with conventional prostatic adenocarcinoma, SCC of the prostate is characterized by an aggressive clinical course, a tendency toward a locally advanced presentation, and an overall poor prognosis [3]. Histologically, these tumors lack glandular differentiation and prostate-specific markers, such as prostate-specific antigen (PSA), but express squamous markers, including p63 and high-molecular-weight cytokeratin [4]. Consequently, serum PSA is often normal or minimally elevated, limiting its utility for diagnosis and disease monitoring and further complicating clinical decision-making [5,6,7].

Prostatic SCC often occurs in the context of prior treatment for adenocarcinoma, including androgen deprivation therapy (ADT) and radiotherapy, raising the possibility of treatment-induced squamous differentiation [7,8,9,10]. In contrast, primary SCC arising de novo in the prostate is rare [11,12]. Although the etiology of de novo prostatic SCC remains unclear, several factors have been proposed to contribute to its development, including chronic inflammation, prior infection, squamous metaplasia, and possible genetic or environmental influences [10,13,14]. However, definitive risk factors have not been clearly established due to the rarity of this entity. Moreover, distinguishing primary prostatic SCC from tumors originating in adjacent structures, such as the bladder or prostatic urethra, remains a diagnostic challenge, particularly in cases of locally advanced disease [15].

Given the absence of standardized management strategies, treatment of primary SCC of the prostate is often individualized based on disease extent, patient comorbidities, and institutional experience. Surgical resection may be considered in select localized cases; however, many patients present with bulky or locally advanced tumors that are not amenable to surgery [11]. Although systemic chemotherapy has been reported, its efficacy remains uncertain, and treatment tolerance may be limited in patients with significant comorbid conditions [13].

Radiotherapy may play a pivotal role in the management of prostatic SCC, particularly in patients with locally advanced disease who are not candidates for surgical resection or systemic chemotherapy [2]. Beyond its established role in symptom palliation, radiotherapy may offer meaningful tumor reduction and short-term locoregional control in selected cases of aggressive malignancy with limited therapeutic alternatives [16]. However, the clinical indications, optimal dose and fractionation, and expected outcomes of radiotherapy in this rare entity remain largely undefined, owing to the scarcity of reported cases [11,16,17].

This study describes de novo primary SCC of the prostate treated with radiotherapy, which resulted in a significant local tumor response. In this case report, we aimed to critically discuss the clinical rationale for selecting radiotherapy, the therapeutic challenges encountered during treatment, and the implications of radiotherapy-related outcomes and complications. This report seeks to contribute to the limited body of evidence and suggests that radiotherapy may represent a reasonable locoregional treatment option in carefully selected patients with prostatic SCC.

## 2. Detailed Case Description

A 56-year-old male was referred from a local medical clinic for evaluation of gross hematuria. The patient’s medical history included acute myocardial infarction treated with coronary artery bypass grafting, end-stage chronic kidney disease requiring maintenance hemodialysis, and long-standing hypertension managed with antihypertensive therapy. Additionally, the patient was an ex-smoker with a smoking history of 30 pack-years and had ceased smoking 10 years before presentation. The patient reported no habitual alcohol consumption. A positive family history of prostate cancer was noted, with a brother previously diagnosed with prostate carcinoma.

**Figure 1:**
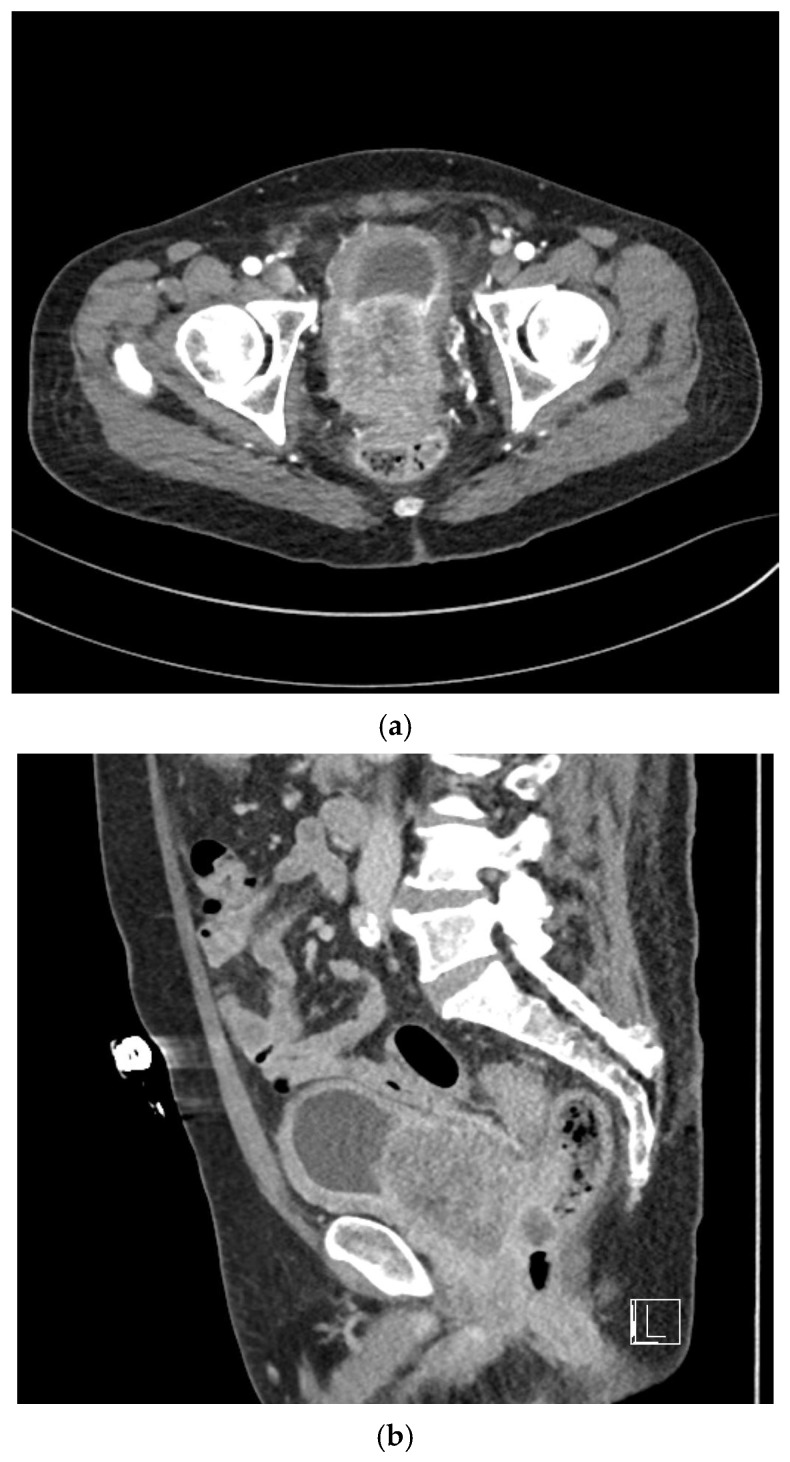

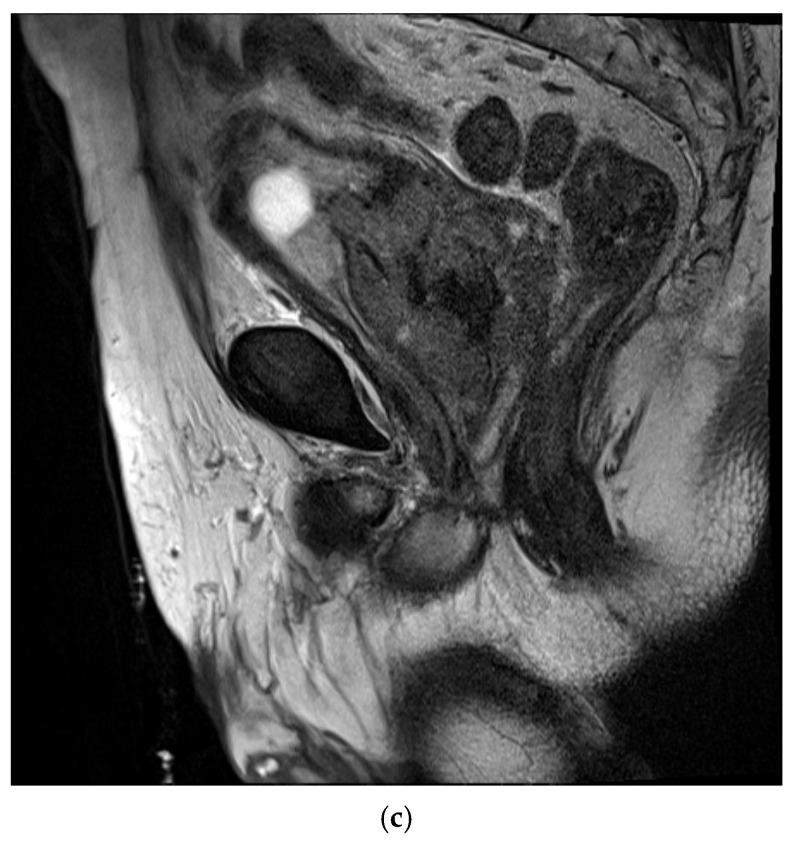
Baseline radiologic findings demonstrating locally advanced prostate squamous cell carcinoma. (**a**) Axial contrast-enhanced computed tomography (CT) image showing asymmetric enlargement of the prostate with heterogeneous enhancement and direct invasion of the urinary bladder. (**b**) Sagittal contrast-enhanced CT image showing diffuse prostatic tumor extension with bladder and seminal vesicle invasion. (**c**) Multiparametric magnetic resonance imaging (MRI) revealing diffuse prostate tumor infiltration with bladder and seminal vesicle invasion and close abutment to the anterior rectal wall.

**Figure 2:**
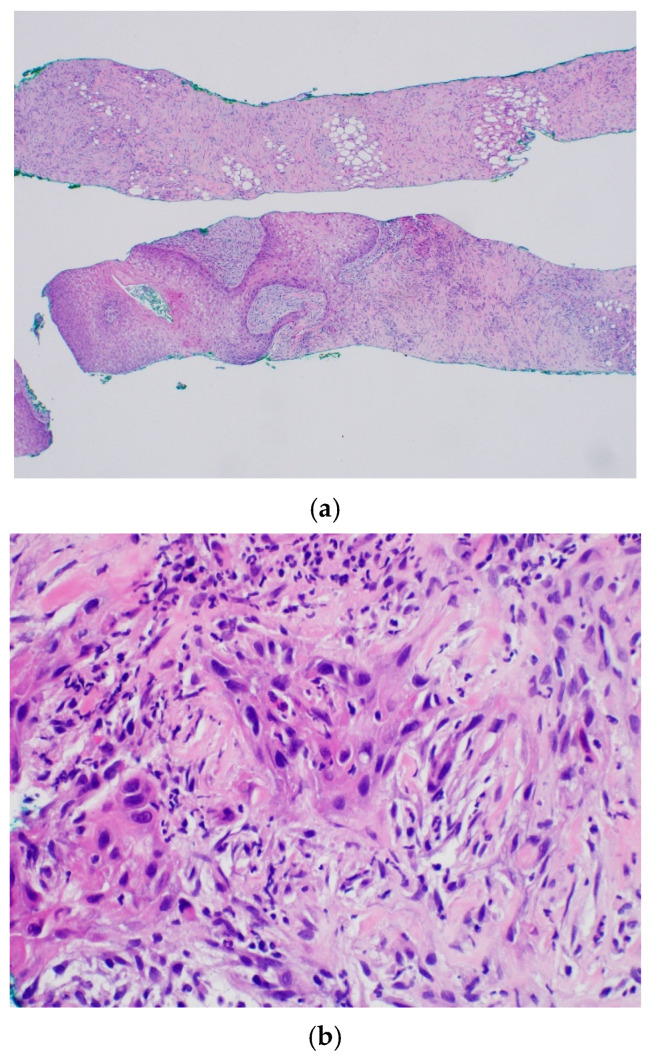
Histopathologic findings of primary squamous cell carcinoma of the prostate. (**a**) Needle core biopsy of the prostate showing squamous cell carcinoma (hematoxylin and eosin [H&E] stain, 40× magnification). The image shows infiltrative nests of malignant squamous cells characterized by abundant eosinophilic cytoplasm and focal keratinization within a dense fibrocollagenous stroma. (**b**) High-power magnification (400×) of the prostatic needle biopsy (H&E stain) shown in (**a**). The image demonstrates invasive squamous cell carcinoma with marked cytologic atypia. Tumor cells display enlarged, pleomorphic, hyperchromatic nuclei and abundant dense eosinophilic cytoplasm. Prominent individual cell keratinization (dyskeratosis) is evident, confirming squamous differentiation. The neoplastic cells are infiltrating into a desmoplastic stroma with associated chronic inflammatory cells.

Urinalysis at presentation demonstrated marked hematuria accompanied by blood clots. Laboratory investigations, including complete blood count and PSA levels, were obtained, revealing an initial PSA level of 0.6 ng/mL. Contrast-enhanced abdominal computed tomography (CT) revealed asymmetric enlargement of the prostate gland with heterogeneous contrast enhancement (Figure 1a,b). Histopathological examination of transrectal ultrasound-guided prostate biopsy specimens demonstrated SCC involving both prostatic lobes (Figure 2). Immunohistochemical analysis demonstrated negative staining for Alpha-Methylacyl-CoA Racemase (AMACR) and positive expression of high-molecular-weight cytokeratin (HMW-CK) and p63. Cystoscopic evaluation revealed no visible lesions or abnormal findings involving the urethra or urinary bladder.

For comprehensive staging, further imaging studies were performed, including multiparametric prostate magnetic resonance imaging (MRI), chest CT, whole-body bone scintigraphy, and positron emission tomography–computed tomography (PET-CT). Prostate MRI demonstrated diffuse prostate cancer with direct invasion of the bladder and seminal vesicles, with close abutment to the rectum (Figure 1c). Bilateral metastatic lymphadenopathy was identified along both pelvic walls. Whole-body bone scintigraphy and PET-CT revealed no evidence of distant metastases to the bone or other organs.

Based on the integrated clinical, radiologic, and pathological findings, the disease was staged as cT4N1M0. The case was reviewed in a multidisciplinary tumor board, and a multimodal treatment strategy was adopted. The patient subsequently underwent definitive pelvic radiotherapy in combination with ADT, consisting of a luteinizing hormone-releasing hormone (LHRH) agonist (goserelin) and an androgen receptor antagonist (bicalutamide).

Before the simulation CT and daily radiotherapy, rectal ballooning was performed according to the institutional protocol. The simulation CT was performed with a slice thickness of 3 mm. Bladder preparation was not performed by drinking a predetermined volume of water because the patient was undergoing maintenance hemodialysis. For radiotherapy planning, the clinical target volume (CTV) was delineated according to the Radiation Therapy Oncology Group (RTOG) guidelines [18]. The superior border of the pelvic CTV was defined at the L5–S1 interspace, and the common, external, and internal iliac nodal regions were included. A 7 mm margin around the pelvic vessels was adopted to generate the pelvic nodal CTV. The planning target volume (PTV) was defined as an isotropic expansion 5 mm from the CTV, except for a reduced posterior margin of 3 mm.

**Figure 3:**
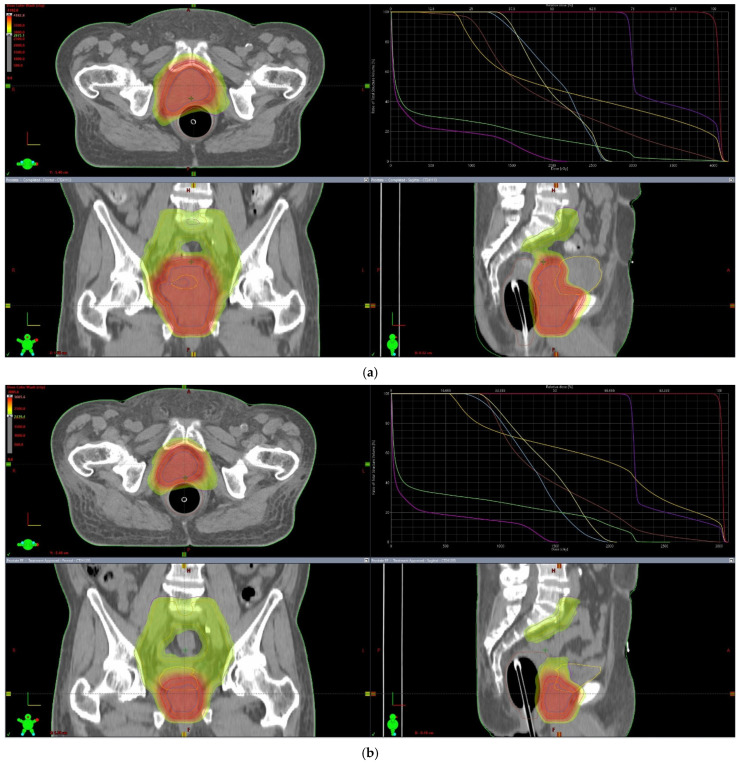
Definitive whole-pelvis radiotherapy with simultaneous integrated boost and adaptive replanning. (**a**) Initial volumetric modulated arc therapy (VMAT) plan delivering 40 Gy in 16 fractions using a simultaneous integrated boost technique. The prostate (red) was prescribed 2.5 Gy per fraction toward 70 Gy, and the elective pelvic lymph nodes (yellow-green) received 1.8 Gy per fraction toward 50.4 Gy. Representative axial, coronal, and sagittal views and dose–volume histograms are shown. (**b**) Adaptive replanning after 40 Gy, followed by delivery of the remaining 30 Gy in 12 fractions to complete the planned doses (70 Gy to the prostate and 50.4 Gy to the pelvic lymph nodes). Re-simulation was performed to account for anatomical changes and to maintain target coverage while optimizing organ-at-risk sparing.

Daily image guidance with cone-beam CT (CBCT) was performed before each treatment fraction, and couch shifts were applied as necessary based on soft-tissue alignment. The patient underwent definitive whole-pelvis radiotherapy using a volumetric modulated arc therapy (VMAT) technique to achieve durable locoregional disease control. A total dose of 70 Gy was delivered in 28 fractions using whole-pelvis radiotherapy with a simultaneous integrated boost (SIB) technique. The prostate received 2.5 Gy per fraction for a total dose of 70 Gy, whereas the elective pelvic lymph node regions received 1.8 Gy per fraction for a total dose of 50.4 Gy within the same overall treatment course. After delivery of 40 Gy in 16 fractions, repeat simulation CT was performed for adaptive replanning to account for anatomical changes during treatment, improve setup reproducibility, and further optimize the sparing of at-risk organs at risk (OARs). Based on the updated plan, the remaining 30 Gy was delivered in 12 fractions to complete the prescribed SIB dose for each target volume (Figure 3). The patient completed the planned course of definitive pelvic radiotherapy combined with ADT. No clinically significant acute gastrointestinal or genitourinary toxicities were observed during radiotherapy.

**Figure 4:**
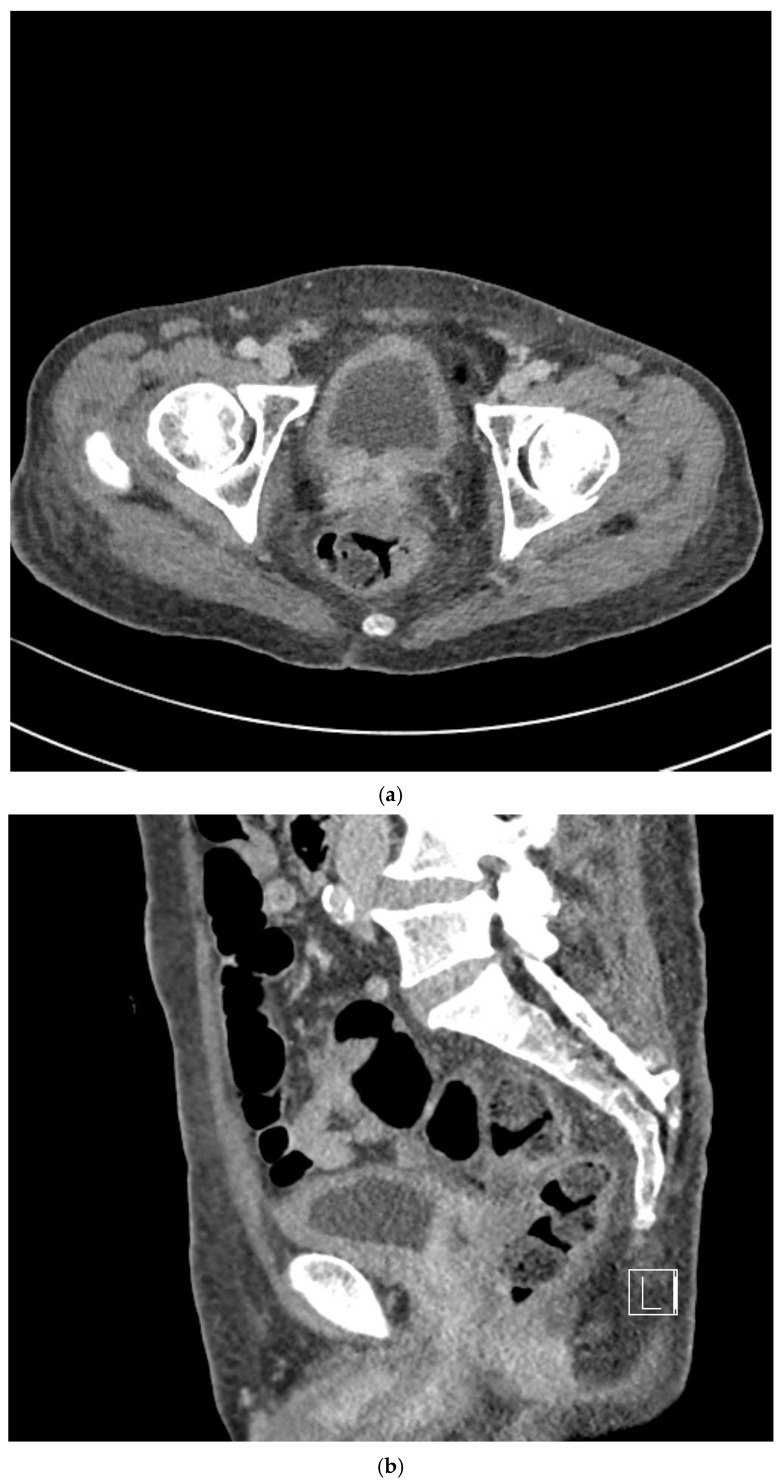

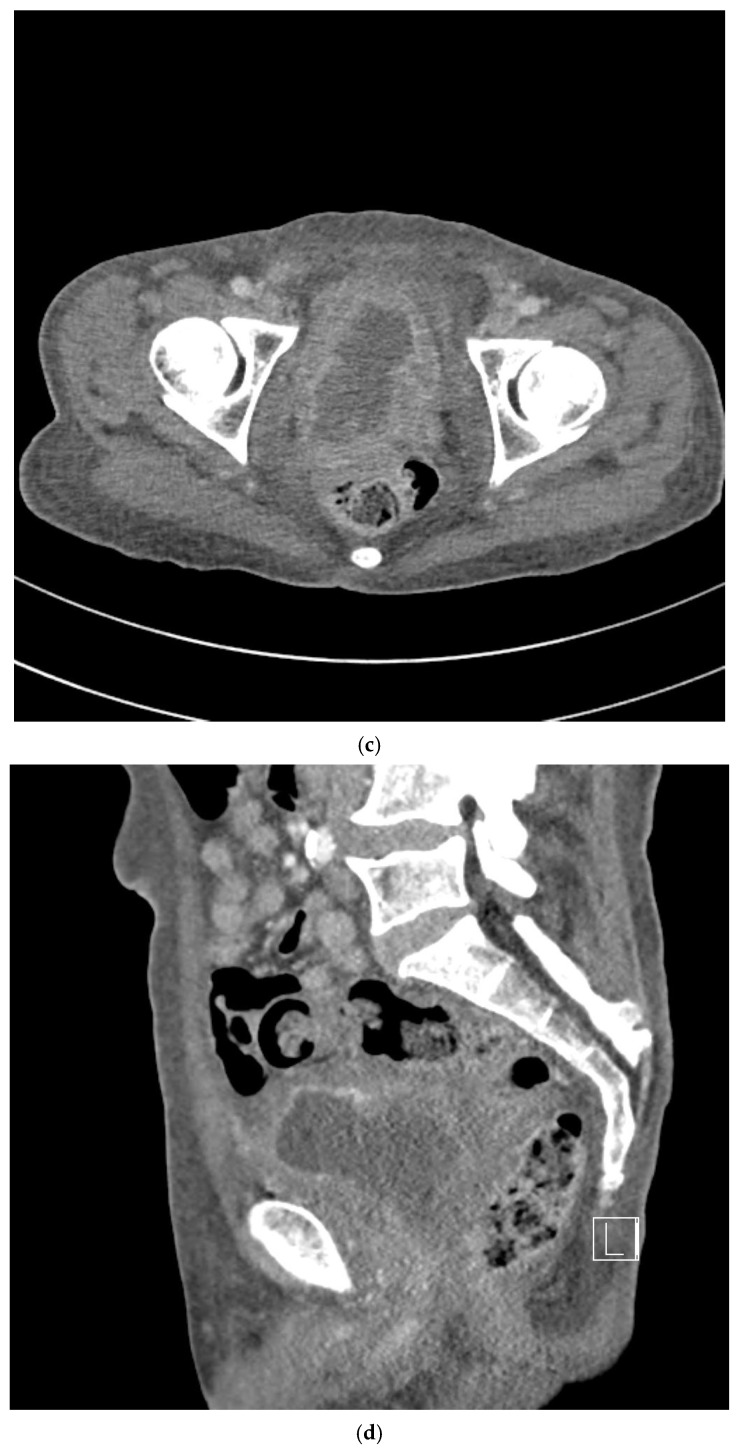

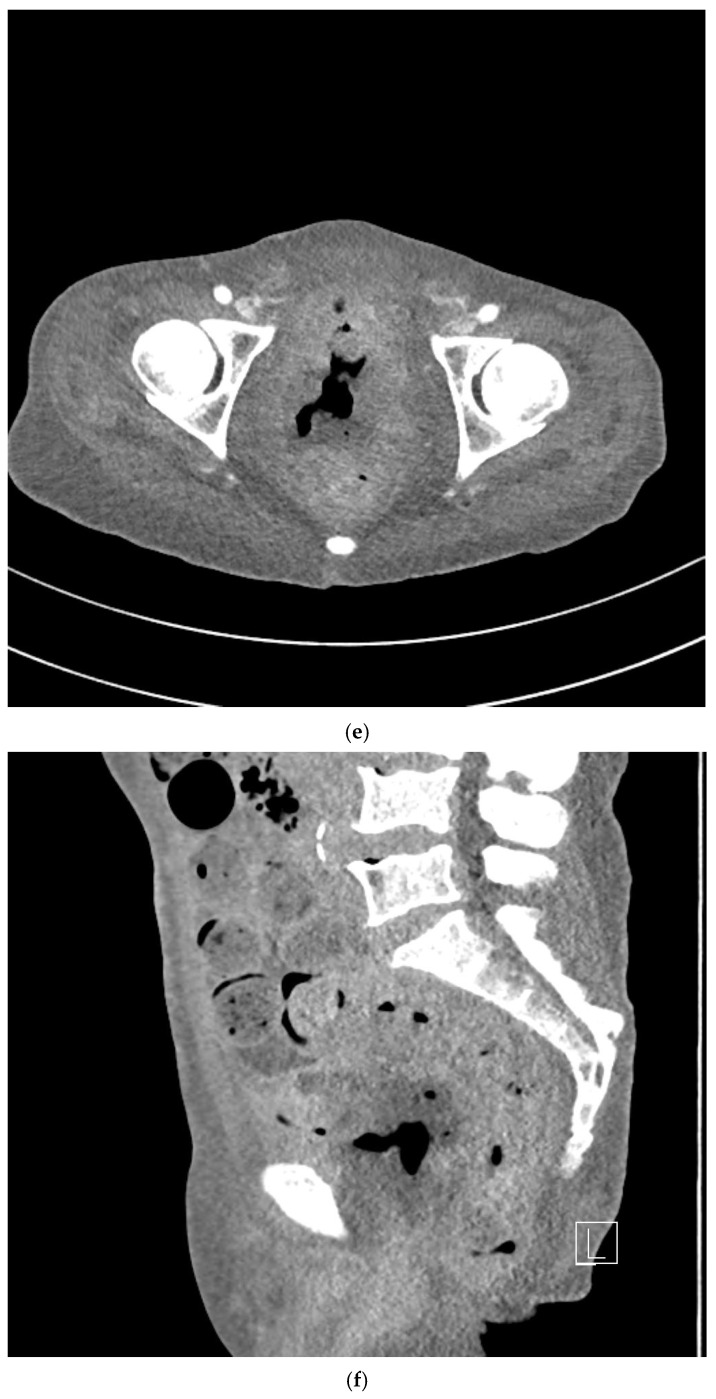
Serial computed tomography (CT) findings following definitive radiotherapy demonstrating tumor regression and subsequent necrosis with fistulous formation. (**a**,**b**) Contrast-enhanced CT images obtained one month after radiotherapy completion demonstrating a marked reduction in prostate tumor size, consistent with a significant treatment response. (**c**,**d**) Follow-up CT images obtained four months after radiotherapy completion showing extensive post-radiotherapy necrosis within the prostate and fistulous communication between the necrotic prostatic cavity and urinary bladder. (**e**,**f**) CT images obtained seven months after radiotherapy completion demonstrating persistent necrosis with maintained fistulous communication.

On follow-up CT performed one month after radiotherapy completion, the prostate mass demonstrated a marked reduction in size, consistent with a significant radiologic response (Figure 4a,b). The PSA level measured at that time was <0.01 ng/mL. On subsequent CT scans obtained four months after radiotherapy, imaging findings were consistent with post-radiotherapy necrosis accompanied by fistulous communication between the necrotic prostatic cavity and urinary bladder, whereas PSA levels remained low (<0.01 ng/mL), although the clinical relevance of PSA monitoring in pure squamous histology remains limited (Figure 4c,d). CT imaging obtained seven months after radiotherapy showed persistent necrosis with maintained fistulous communication (Figure 4e,f).

At the last follow-up, seven months after radiotherapy completion, the patient was transferred to a local convalescent hospital for supportive care because of a decline in general condition. The overall clinical course, including treatment response and subsequent complications, is summarized in Table 1.

## 3. Discussion

This study describes de novo primary SCC of the prostate presenting as locally advanced disease with bladder invasion and pelvic nodal involvement in a medically inoperable patient with substantial comorbidities. Given the contraindication to systemic chemotherapy, radiotherapy was selected as the primary definitive treatment modality. The marked tumor regression observed following radiotherapy highlights its potential role in achieving meaningful locoregional control in this challenging clinical setting.

### 3.1. Biological and Diagnostic Considerations

De novo primary SCC of the prostate is exceedingly rare, and its biological origin remains poorly understood [1]. Most reported cases occur as secondary transformation following prior treatment for conventional adenocarcinoma, particularly after ADT or radiotherapy, suggesting treatment-induced squamous differentiation [7,8,9,10].

In contrast, de novo primary SCC arising in the absence of prior prostate-directed therapy is uncommon and may represent a biologically distinct entity [15]. The proposed mechanisms include malignant transformation of basal epithelial cells, squamous metaplasia related to chronic inflammation, and aberrant differentiation of pluripotent progenitor cells, suggesting potential biological distinctions from conventional adenocarcinoma [14,19].

Unlike adenocarcinomas, SCCs generally do not produce clinically meaningful PSA, rendering PSA-based screening and treatment monitoring inherently unreliable [13]. Radiologic findings are often nonspecific, particularly in locally advanced disease with adjacent organ invasion, making differentiation from tumors arising in adjacent organs challenging [13]. In cases with bladder invasion, determining the primary site using imaging alone can be particularly challenging [15].

Therefore, immunohistochemistry is essential for diagnosis, with typical expression of p63 and HMW-CK in the absence of glandular markers such as AMACR [1]. In the patient described here, the absence of prior prostate-directed therapy and the histopathologic confirmation of pure squamous differentiation strongly supported the diagnosis of de novo primary SCC, effectively excluding treatment-induced transformation and reinforcing the biological distinctiveness of this entity.

### 3.2. Therapeutic Decision-Making and the Central Role of Radiotherapy in Medically Inoperable Prostatic SCC

The optimal management of primary SCC of the prostate remains unclear owing to its extreme rarity and the absence of prospective clinical evidence [13]. Most available data are derived from case reports or small retrospective series, and treatment strategies are largely extrapolated from conventional prostatic adenocarcinoma or squamous malignancies of other organs [20,21]. Consequently, treatment decisions must be individualized to balance the theoretical oncological benefits against patient-specific feasibility.

Given the aggressive biological behavior of prostatic SCC and its frequent presentation as bulky, locally invasive disease, multimodal therapy has often been advocated when feasible [1,11]. Surgical resection may be considered in select patients with organ-confined tumors; however, true surgical candidates are uncommon [1,10]. Many patients present with invasion of adjacent structures such as the bladder, seminal vesicles, or rectum, rendering radical surgery technically challenging and potentially morbid [13]. Moreover, surgery alone rarely achieves durable disease control, and early distant progression can occur even after complete resection [22].

Therefore, combined chemoradiotherapy (CCRT) has emerged as a potentially effective strategy for the treatment of locally advanced disease [11,16]. Platinum-based regimens, commonly incorporating cisplatin, 5-fluorouracil, or mitomycin C, have been administered concurrently with radiotherapy, based on established chemoradiotherapy paradigms in other SCCs such as anal, cervical, and head and neck cancers [23,24,25]. Selected cases have shown complete response and medium-term remission [1,16]. Although the number of reported patients remains small and the treatment regimens are heterogeneous, these observations suggest that systemic therapy may enhance locoregional control in biologically aggressive SCC [1,11,16].

However, the feasibility of multimodal therapy is highly dependent on comorbidities. Platinum-based chemotherapy carries significant risks, including nephrotoxicity and systemic toxicity, which may preclude its safe administration in medically fragile individuals [26]. In patients with severe renal impairment requiring maintenance hemodialysis, cisplatin-based chemotherapy is often impractical. Curative surgery may also be infeasible in the presence of cT4 disease with direct bladder invasion and bilateral pelvic nodal involvement. Thus, although multimodal therapy may represent an ideal strategy for fit patients, it cannot be operationalized in certain clinical contexts.

Therefore, radiotherapy has emerged not as an adjunct to systemic treatment but as the only viable modality to deliver definitive-intent locoregional therapy. This shift reframes the therapeutic question. Rather than evaluating the incremental contribution of radiotherapy within a multimodal regimen, the more relevant issue becomes whether definitive-dose radiotherapy alone can achieve meaningful tumor control when other curative intent options are contraindicated.

The role of ADT in the treatment of prostatic SCC remains controversial. Pure squamous histology is generally considered androgen receptor (AR)-negative and is biologically distinct from conventional adenocarcinoma, suggesting that ADT is unlikely to exert substantial direct antitumor effects [10,22]. Nevertheless, complete exclusion of an occult adenocarcinomatous component may be challenging in needle biopsy specimens, as sampling limitations may underestimate mixed histology, including adenosquamous differentiation [6]. In addition, limited reports have described heterogeneous AR expression in rare cases of prostate SCC [1,14].

In the present case, given the locally advanced, node-positive disease and the absence of established systemic treatment standards, ADT was administered as a precautionary adjunct rather than as a biologically targeted therapy. The primary definitive modality remained radiotherapy, while ADT was intended to provide potential systemic coverage in the setting of diagnostic uncertainty. Although PSA levels remained suppressed during follow-up, the interpretive value of PSA in pure squamous histology is inherently limited and should be regarded as supplementary rather than definitive evidence of disease control. Accordingly, the clinical response observed in this case should be primarily interpreted in the context of definitive-dose radiotherapy rather than hormonal manipulation. Therefore, ADT should be regarded as a precautionary adjunct rather than an essential component of treatment in this context. From a clinical decision-making perspective, the addition of ADT was considered a low-risk intervention with potential systemic coverage, whereas omission of systemic therapy could risk undertreatment in the setting of possible mixed histology.

### 3.3. Radiotherapy as a Central Modality in Prostatic SCC

Squamous tumors are generally considered more radiosensitive than conventional prostatic adenocarcinomas [22]. The marked tumor regression observed one month after radiotherapy supports the biological plausibility of clinically meaningful radiosensitivity. In contrast to PSA dynamics, which are unreliable in pure SCC, radiographic findings provide more robust evidence of treatment response.

Compared with previously reported de novo primary prostatic SCC cases treated with radiotherapy, the most durable remissions have been described in the setting of combined chemoradiotherapy (Table 2) [11,16,17,27,28,29]. Long-term disease control extending beyond two years has been reported in selected patients receiving concurrent platinum-based regimens [11,17]. By contrast, evidence regarding radiotherapy-dominant or radiotherapy-alone strategies remains limited, and the long-term durability of tumor control without systemic chemotherapy is uncertain [1,22]. The observations in the present study do not resolve this uncertainty, particularly given the relatively short follow-up period resulting from non-oncologic clinical deterioration. Nevertheless, this case suggests that definitive-dose radiotherapy alone may induce substantial tumor regression in medically inoperable patients with node-positive, locally advanced disease. However, the limited duration of follow-up precludes definitive conclusions regarding long-term oncological durability.

The radiotherapy strategy, employing whole-pelvis irradiation with an SIB technique and adaptive replanning, was extrapolated from high-risk adenocarcinoma protocols, given the absence of SCC-specific dose recommendations. Dose intensification was selected because of the bulky T4 disease and nodal involvement, reflecting a pragmatic approach grounded in a biological rationale. This experience underscores a broader challenge in rare malignancies: treatment paradigms often rely on rational extrapolation rather than disease-specific evidence.

Importantly, the marked tumor regression observed after radiotherapy was accompanied by extensive posttreatment necrosis and subsequent fistulous communication between the prostate and bladder. This complication likely reflects the interaction between rapid tumor regression and preexisting bladder invasion in patients with bulky T4 disease. The mechanisms and clinical implications of this complication are discussed below:

Collectively, this case suggests that definitive radiotherapy may represent a feasible locoregional treatment option in selected medically inoperable patients with de novo primary prostatic SCC. Although multimodal therapy may offer the greatest theoretical potential for durable control, real-world clinical constraints frequently require individualized treatment strategies. In such contexts, definitive-dose radiotherapy may serve as the principal therapeutic modality and can induce substantial early tumor regression, even in node-positive, locally advanced presentations. However, the durability of radiotherapy alone remains uncertain and warrants further investigation in larger cohorts.

### 3.4. Necrosis and Fistula Formation After Rapid Tumor Regression

Despite marked tumor regression, post-radiotherapy imaging revealed extensive tumor necrosis, accompanied by fistulous communication between the prostate and urinary bladder. Such findings likely reflect a multifactorial process rather than isolated radiation toxicity [30,31].

The tumor demonstrated direct bladder invasion at baseline (cT4), indicating preexisting disruption of the vesicoprostatic interface. Although radiation exposure may contribute to tissue breakdown, direct bladder invasion may predispose the vesicoprostatic interface to structural compromise [31,32]. Rapid necrosis in radiosensitive, locally invasive SCC may further destabilize already disrupted tissue planes [33]. Delivery of definitive-dose radiotherapy (70 Gy) to a bulky, radiosensitive squamous malignancy may therefore induce rapid central necrosis, further weakening already compromised tissue planes and predisposing to fistulous breakdown.

Similar necrosis-related fistulous complications have been described in other radiosensitive cell carcinomas, including anal and cervical SCC, particularly when tumors invade adjacent hollow organs [31,34]. These observations suggest that rapid tumor regression in highly radiosensitive SCC may paradoxically increase the risk of structural failure in locally invasive diseases.

These observations underscore the importance of careful pretreatment counseling and vigilant posttreatment monitoring when definitive-dose radiotherapy is delivered in the setting of adjacent organ invasion.

### 3.5. Clinical Implications and Limitations

These observations suggest that definitive-dose radiotherapy represents a feasible option that may be associated with early tumor regression in medically inoperable patients when chemotherapy is contraindicated. In the context of locally advanced disease, tumor reduction may also contribute to symptomatic improvement, suggesting that radiotherapy may serve as a meaningful locoregional strategy when alternative treatment options are limited. However, this case also illustrates the complexity of the treatment response, as rapid tumor necrosis was associated with significant complications, underscoring the importance of careful risk–benefit assessment. Therefore, treatment decisions should extend beyond anticipated oncological responses and incorporate patient comorbidities, life expectancy, and quality-of-life considerations.

This study has some limitations. Because these findings derive from a single clinical observation, they are not generalizable, and the relatively short follow-up limits assessment of long-term oncologic outcomes, including local control, late toxicity, and survival outcomes. Despite these limitations, this case provides clinically relevant insights into the potential role of radiotherapy in de novo primary prostatic SCC and highlights the importance of individualized, multidisciplinary decision-making in the absence of established treatment guidelines.

## 4. Conclusions

De novo primary SCC of the prostate is an exceptionally rare and biologically aggressive malignancy characterized by low PSA expression, advanced local presentation, and the absence of standardized treatment guidelines. Therefore, management is inherently individualized, particularly in patients with significant comorbidities that preclude multimodal therapy.

In the present case, definitive-dose whole-pelvis radiotherapy achieved marked early tumor regression in a medically inoperable patient with locally advanced, node-positive disease. Although long-term oncologic durability could not be fully evaluated due to non-oncologic clinical deterioration related to underlying comorbid conditions, sustained radiologic regression during the available follow-up period and the absence of radiologic progression suggest that radiotherapy may have biological activity and clinical feasibility as a principal locoregional strategy in carefully selected patients.

Given the scarcity of reported de novo cases and the absence of established treatment standards, this case suggests that definitive radiotherapy may induce substantial early tumor regression in carefully selected medically inoperable patients with de novo prostatic SCC, warranting individualized multidisciplinary consideration in this rare entity.

## Figures and Tables

**Table 1:** Timeline of treatment, radiographic response, and post-radiotherapy complications.

Time Point	Key Clinical Events	PSA (ng/mL)
Initial diagnosis (baseline)	Gross hematuria; imaging revealed a huge prostate mass	0.6
ADT + radiotherapy (~1 months after diagnosis)	ADT initiated Definitive whole-pelvis radiotherapy (70 Gy in 28 fractions, 5.5 weeks)	NA
1 month after radiotherapy (~3 months after diagnosis)	Marked reduction in prostate mass size on follow-up CT	<0.01
4 months after radiotherapy (~6 months after diagnosis)	Post-radiotherapy necrosis with fistulous communication between the prostate and urinary bladder	<0.01
7 months after radiotherapy (~9 months after diagnosis)	Persistent post-radiotherapy necrosis with fistulous communication on follow-up CT Transferred due to non-oncologic clinical decline	<0.01

ADT, androgen deprivation therapy; PSA, prostate-specific antigen; CT, computed tomography.

**Table 2:** Reported cases of de novo primary squamous cell carcinoma of the prostate treated with radiotherapy.

Author (Year)	Stage	Treatment Strategy	RT Dose/Field	Outcome
Uchibayashi et al. (1997) [29]	Organ-confined (reported in review)	RT + bleomycin + cisplatin	Pelvic RT 54 Gy	Favorable local control; survival 21 months
Okada & Kamizaki (2000) [28]	cT3N1M0	RT + peplomycin + cisplatin	Pelvis 50 Gy + prostate 10 Gy	CR for 18 months
Muñoz et al. (2007) [16]	cT3aN0M0	Cisplatin + 5-FU → RT	Pelvis 46 Gy + boost 20 Gy + 6 Gy (total 72 Gy)	CR for 5 years; later recurrence; follow-up 5 years
Biswas et al. (2015) [11]	cT4N1M0	RT + mitomycin C + 5-FU	Pelvis 45 Gy + boost 9 Gy (total 54 Gy)	CR for 27 months
Onoda et al. (2016) [17]	cT3bN1M0	RT + docetaxel + cisplatin + 5-FU	Pelvis + prostate (total 64 Gy)	CR for 24 months
Present case	cT4N1M0	RT + ADT (chemotherapy contraindicated)	pelvis 50.4 Gy & prostate 70 Gy	Marked response; necrosis/fistula; last follow-up 7 months

## Data Availability

The raw data that support the findings of this study are available from the corresponding author upon reasonable request. However, the data are not publicly available due to privacy considerations.

## Abbreviations

The following abbreviations are used in this manuscript:

ADT	Androgen deprivation therapy
AMACR	Alpha-methylacyl-CoA racemase
AR	Androgen receptor
CBCT	Cone-beam computed tomography
CCRT	Concurrent chemoradiotherapy
CT	Computed tomography
CTV	Clinical target volume
GI	Gastrointestinal
GU	Genitourinary
HMW-CK	High-molecular-weight cytokeratin
LHRH	Luteinizing hormone-releasing hormone
MRI	Magnetic resonance imaging
OARs	Organs at risk
PET-CT	Positron emission tomography–computed tomography
PSA	Prostate-specific antigen
PTV	Planning target volume
RTOG	Radiation Therapy Oncology Group
SCC	Squamous cell carcinoma
SIB	Simultaneous integrated boost
VMAT	Volumetric modulated arc therapy
